# Persistent Stress-Induced Neuroplastic Changes in the Locus Coeruleus/Norepinephrine System

**DOI:** 10.1155/2018/1892570

**Published:** 2018-06-13

**Authors:** Olga Borodovitsyna, Neal Joshi, Daniel Chandler

**Affiliations:** Department of Cell Biology and Neuroscience, Rowan University School of Osteopathic Medicine, Stratford, NJ 08084, USA

## Abstract

Neural plasticity plays a critical role in mediating short- and long-term brain responses to environmental stimuli. A major effector of plasticity throughout many regions of the brain is stress. Activation of the locus coeruleus (LC) is a critical step in mediating the neuroendocrine and behavioral limbs of the stress response. During stressor exposure, activation of the hypothalamic-pituitary-adrenal axis promotes release of corticotropin-releasing factor in LC, where its signaling promotes a number of physiological and cellular changes. While the acute effects of stress on LC physiology have been described, its long-term effects are less clear. This review will describe how stress changes LC neuronal physiology, function, and morphology from a genetic, cellular, and neuronal circuitry/transmission perspective. Specifically, we describe morphological changes of LC neurons in response to stressful stimuli and signal transduction pathways underlying them. Also, we will review changes in excitatory glutamatergic synaptic transmission in LC neurons and possible stress-induced modifications of AMPA receptors. This review will also address stress-related behavioral adaptations and specific noradrenergic receptors responsible for them. Finally, we summarize the results of several human studies which suggest a link between stress, altered LC function, and pathogenesis of posttraumatic stress disorder.

## 1. Introduction

Stressful stimuli and events engage a number of brain circuits that ultimately activate the hypothalamic-pituitary-adrenal (HPA) axis. During periods of stress, the paraventricular nucleus of the hypothalamus (PVN) releases the stress peptide corticotropin-releasing factor (CRF), which stimulates both direct central and indirect peripheral effects, activating signal transduction pathways that enhance catabolism of energy stores and mobilize physiological and psychological resources of the organism to permit an appropriate behavioral response to the stressor. These pathways become dysregulated following chronic or traumatic stress, which leads to destabilization of homeostasis and impaired immune, cardiovascular, and gastrointestinal functions, and promoting central nervous system (CNS) changes associated with depressive and anxiety-like behaviors that contribute to the diagnosis of stress-associated disorders [1–10]. The ability to mobilize CNS function to respond to stressful stimuli and ensure survival is explained in part by changes in neuroplastic adaptations. Several CNS structures have been demonstrated to undergo neuroplastic changes following stress [2, 11–24] which may contribute to stress-associated anxiety and mood disorders [12, 14, 19, 25, 26]. Chronically altered noradrenergic transmission is a characteristic of many neuropsychiatric and neurodegenerative disorders [12, 27–34], and therefore, short- and long-term stress-induced adaptations in norepinephrine- (NE-) containing cell bodies may contribute to these conditions. For the purposes of this review, we consider short-term effects to refer to the immediate and primary action CRF signaling during stressor exposure and the stress response on electrophysiological properties such as membrane depolarization and action potential generation that result from the opening of channels already inserted in the membrane. Long-term effects on the other hand include persistent changes that continue long after the stress response and CRF signaling have ceased and resulted from intracellular signaling cascades that promote receptor and channel trafficking, altered gene expression, and neurite outgrowth.

**Figure 1:**
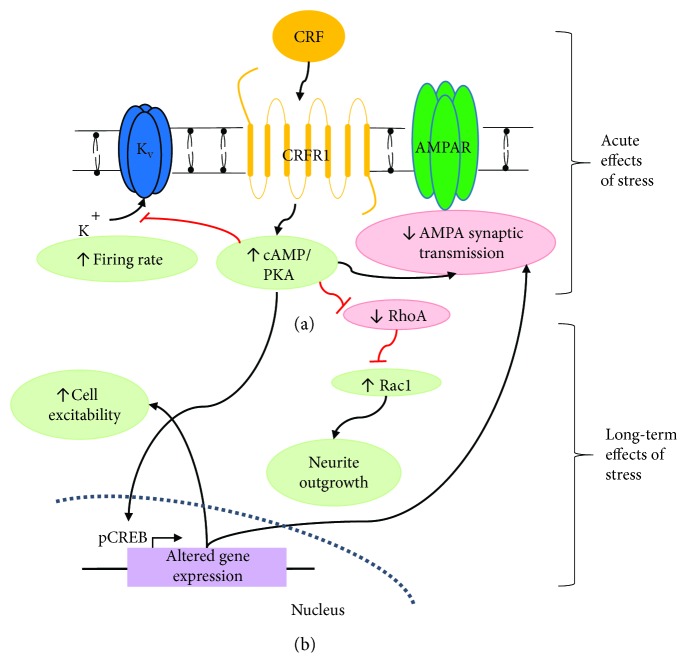
Model of signal transduction pathways induced by stress in LC neurons. (a) Pathways which mediate short-term effects of stressor exposure. CRF interacts with CRFR1, which through Gs-coupled receptor mechanisms increases intracellular cAMP levels, reducing potassium conductance resulting in cell depolarization. Through unknown mechanisms, CRF decreases glutamatergic synaptic transmission through AMPARs. (b) Pathways which mediate long-term effects of stressor. Initial CRF activation of Gs-coupled CRFR1 increases PKA activity, which phosphorylates CREB to initiate expression of stress-induced genes. These could potentially include genes regulating AMPAR and voltage-gated ion channel expression. Inactivation of RhoA by PKA phosphorylation disinhibits Rac1 to increase neurite outgrowth via actin remodeling and microtubule stabilization.

A major node in the stress response that promotes noradrenergic signaling in the CNS is the brain stem nucleus locus coeruleus (LC). The LC and other smaller noradrenergic brainstem nuclei, such as A1/C2 region in the solitary tract, are activated by CRF and reciprocally communicate with the HPA axis. Activation of A1/C2 promotes a positive feedback loop in stress circuitry by releasing NE in the PVN which stimulates CRF production and release by engaging *α*_1_-adrenoreceptors (*α*_1_AR) [35, 36]. The LC is the primary source of NE to the forebrain [37–45], where its actions affect sleep/wake cycles, sensory signal discrimination and detection, and cognition [2, 37, 46, 47]. It is innervated by a number of stress-responsive CRF-containing brain regions which when released, acts on CRF receptor 1 (CRFR1) receptor to produce acute changes in LC physiology and responsiveness to synaptically released transmitters [48–51]. Additionally, activation of CRFR1 stimulates Gs proteins and cAMP production [48, 52], which promotes numerous genetic and cellular effects [28, 50, 53–57]. These observations suggest that LC neurons may undergo many long-lasting stress-induced adaptations (Figure 1). These changes include receptor trafficking [58–60], altered expression of genes necessary for transmitter synthesis and release [28, 54–56, 61], protein kinases that activate transcription factors [57] and growth factors [18], electrophysiological properties [53, 62], and morphological changes [50, 53, 63], all of which would directly impact LC function at both immediate and chronic time point poststress.

While most investigations have focused on the transient effects of stress and CRF on LC function [48, 49, 51, 64–66], some have examined their lasting impact [28, 50, 52–54, 56, 57, 62]. This review will summarize how stress and CRF signaling persistently modif**y** morphological and physiological features of the locus coeruleus/norepinephrine (LC/NE) system and its associated behaviors from a genetic, cellular, and neuronal circuitry/transmission perspective. While the stress-induced plastic changes that occur in LC and other brain regions during disease pathogenesis are not entirely clear, identifying how stress can chronically alter the function of this broadly projecting brainstem nucleus across multiple levels of regulation represents an important step forward in clarifying the mechanisms of conditions characterized by hyperactive noradrenergic transmission.

## 2. LC/NE Synaptic Plasticity Changes during Stress

### 2.1. Adaptive Functional and Anatomical Changes of LC after Stress

HPA axis activation is pivotal for mediating the central stress response. Through the release of peripheral and central neurohormones, it mobilizes various body tissues and brain areas to orchestrate an appropriate physiological and behavioral response. Importantly, during stressor exposure, CRF is released onto the LC by the PVN and other CRF-containing stress-responsive structures, such as the bed nucleus of the stria terminalis, Barrington's nucleus, and the central nucleus of the amygdala [67–73] which increase its tonic discharge [51, 62, 65, 66]. LC activity correlates highly with an animal's behavioral state: during quiet rest, LC discharges slowly in a highly regular fashion. During periods of focused attention, a phasic mode of operation dominates such that LC responds to salient stimuli with high-frequency bursts of action potentials that facilitate orientation and sustained attention towards behaviorally relevant stimuli [74]. During stress, CRF causes increased tonic discharge which compromises the ability of LC to respond to salient sensory stimuli with phasic bursts. This leads to impairments in sensory signal discrimination, several aspects of cognition, and a generally anxious state [2, 37, 74–77]. While this might seem generally maladaptive, a consequence of short-term stress-induced LC activation is to promote behaviors that increase the likelihood of survival in a threatening situation [2, 66]. By increasing LC discharge [51, 62, 65, 66] and therefore forebrain NE release [78–82], prefrontal cortical operations are inhibited [3, 78], promoting a behavioral phenotype characterized by broad scanning attention and vigilance [2, 66, 81, 83], which facilitates escape from a threatening situation.

The role of LC in stress has been the subject of study since 1970, when karyometric studies of sleep-resistant rabbits demonstrated an increase in nuclear size during stress [84]. Subsequently, an extensive body of literature has shown that LC is critical for mediating stress-induced behavioral and neuroendocrine responses. The electrophysiological effects of stress and CRF on LC have been well characterized in a number of *in vivo* and ex vivo studies [48, 49, 51, 53, 62, 65, 85]. *In vivo*, CRF increases tonic/spontaneous LC discharge [65, 86, 87] through a cyclic AMP (cAMP)/protein kinase A-dependent mechanism that depolarizes the membrane by decreasing potassium conductance [48]. Additionally, CRF has been demonstrated to decrease sensory-evoked phasic responses by LC [65, 86]. This effect could partially be explained by recent findings from our laboratory that show that a high concentration of bath-applied CRF [49] and preexposure to acute stress [62] both diminish excitatory glutamatergic synaptic transmission in LC. We found that these electrophysiological effects persist for at least a week poststress in adolescent rats. Moreover, electrophysiological changes which were absent immediately after stressor exposure develop over seven days, including increased intrinsic excitability and a hyperpolarized threshold for action potential generation [62]. These findings suggest that LC cells in adolescent rat brain undergo long-lasting changes following even short-term acute stressor exposure and lead to chronically increased forebrain NE concentration and behavioral changes.

### 2.2. CRF and Morphological Changes

CRF orchestrates a series of neuroplastic changes in LC neurons and LC-derived cell cultures [50, 52, 53, 58, 63]. Specifically, CRF triggers morphological changes in immortalized catecholaminergic neurons, such as the formation of long neurites with prominent growth cones [52]. Similarly, another study demonstrated the ability of CRF to promote neuronal outgrowth in organotypic slice cultures of rat LC [50]. In this study, it was found that 12 hours of CRF exposure increased the number of primary processes and branching pattern of neurites. Mechanisms of dendritic growth regulation by CRF have been proposed to occur through Rac and RhoA GTPases (Figure 1), which regulate intracellular actin dynamics and spine length [88]. Elsewhere in the brain, inhibition of Rac1 has been shown to promote strong effects on dendritic spines from apical and basal dendrites on pyramidal neurons with relative absence of branching effects [89].

**Figure 2:**
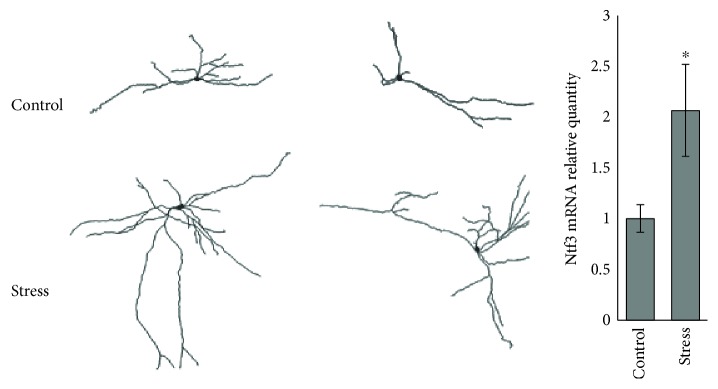
LC neurons from stressor-exposed animals show a trend for increased dendritic complexity. Representative traced neurons from control (top) and stressor exposed (bottom) animals filled with biocytin reveal a tendency for LC cells from stressed rats to possess larger and more complex dendritic arbors a week after stressor exposure. Additionally, neurotrophin 3, which promotes neurite outgrowth and dendritic proliferation, is upregulated in LC one week after stressor exposure. ^∗^*p* < 0.05 versus control.

Additional unpublished observations from our laboratory also suggest that in animals subjected to acute intense stressor exposure, LC cells might undergo morphological changes. We have previously shown that fifteen minutes of combined physical restraint and exposure to predator odor induces a number of long-lasting changes in LC function that are accompanied by chronically increased anxiety-like behavior [62]. During whole-cell patch clamp electrophysiological recordings, some neurons were filled with biocytin so their morphology could be recovered. Preliminary findings show that LC cells from stressed animals have larger and more complex dendritic arbors than those from control rats. Additionally, using RNA-Seq, we identified that expression of Ntf3, the gene for neurotrophin 3, which promotes neuronal survival, differentiation, and neurite outgrowth [18, 90], was approximately twice as high in rats one week after stressor exposure than in their control counterparts (Figure 2). These observations, in combination with earlier reports of stress-induced morphological alterations in LC neurons [50, 52, 53, 75, 91–93], suggest that stress may cause long-lasting changes in noradrenergic transmission throughout the CNS in response to even acute stressor exposure. Such an effect on CNS noradrenergic transmission might be achieved through morphological plasticity because as LC dendrites and axons proliferate, there would be more sites of afferent input to excite LC neurons and a greater density of release points from which NE efflux could occur upon this enhanced excitation, respectively. Such findings could have important implications for posttraumatic stress disorder (PTSD), a condition in which evidence suggests that NE transmission is impaired [12, 31, 79, 94].

It is interesting to note that rodent LC neurons are sexually dimorphic with respect to their morphological characteristics and response to stress/CRF exposure. Female LC dendritic arbors have been reported to extend further into the peri-LC region where synaptic contacts with CRF-positive afferents are made [71, 95, 96] and are larger with more branching points than those of males [68, 97]. This suggests that the female LC might be subjected to greater afferent regulation by CRF and therefore more stress-responsive. This sexual dimorphism might provide a structural basis for differences in emotional arousal between sexes and the greater increased susceptibility of females to anxiety disorders [98]. Interestingly, in mice that genetically overexpress CRF, the complexity of male dendritic morphology increases to resemble the morphology of wild-type and CRF-overexpressing females. This further suggests that enhanced CRF signaling produces neurite proliferation and extension in LC [58]. Such observations provide further evidence for stress and CRF-induced central noradrenergic reorganization.

### 2.3. CRF and Modified AMPA Receptor-Dependent Synaptic Transmission

Plasticity is highly dependent on the AMPA receptor (AMPAR), an ionotropic glutamate receptor, permeable to Na^+^ and Ca^2+^ ions. It is composed of four subunits: GluA1, GluA2, GluA3, and GluA4, which form a heterotetramer. [99–103]. We have previously shown that both stressor exposure *in vivo* [62] and CRF exposure ex vivo [49] alter LC AMPAR signaling. Given that stress and CRF can alter AMPAR-dependent transmission, this receptor might play a critical role in stress-induced neural plasticity. Several mechanisms could account for altered AMPAR functioning in LC following CRF exposure. CRF signaling in LC causes internalization of its own receptor [59, 60, 63], and thus, if CRF and AMPARs are in close apposition to one another on postsynaptic LC membranes, CRF receptor trafficking might inadvertently induce AMPAR internalization as well. This is particularly important with respect to some of the intracellular proteins that AMPARs interact with. AMPARs interact directly and indirectly with kinases and GTPases that regulate actin cytoskeletal dynamics [50, 53, 99–101, 104, 105]. In particular, Rho GTPase activity is modulated by guanine nucleotide exchange factors (GEFs), which are known to interact with *surface*-*expressed* AMPARs and promote synaptic plasticity [104]. Thus, if CRF causes shuttling of vesicular AMPARs to the cell membrane, which, based on our prior observations, might occur during high concentrations of CRF exposure [49], their association with GEFs could promote structural plasticity in LC neurons through interaction with Rho GTPases and modulation of cytoskeletal structure. Identifying the mechanisms that link CRF and AMPA receptor function and trafficking could be informative of how LC cells adapt morphologically following stress, thus providing insights to a number of disease states in which LC plasticity is perturbed [29, 50, 53, 106, 107]. In addition to receptor trafficking and altered gene expression, there are other posttranslational modifications that can be made to the AMPAR and its subunits which could promote plastic changes to LC neurons. Using data from both LC and non-LC studies of AMPAR function and modification, we will review possible mechanisms for AMPAR regulation.

There are multiple mechanisms of posttranslational regulation of AMPAR function, which include reversible phosphorylation, ubiquitination, and palmitoylation [108–110]. CRF stimulates cAMP synthesis and PKA activity [48, 50, 52], and therefore, stress could potentially alter AMPAR phosphorylation states. The GluA1 subunit is phosphorylated at different positions at the C-terminal end. For example, phosphorylation at Ser-845 by PKA [101] and Ser-831 by PKC and CaMKII [111, 112] increase single channel conductance. Phosphorylation of Ser-818 and Thr–840 by PKC increases the mean channel conductance [113]. Importantly, mechanisms that increase channel conductance have been shown to promote activity-dependent plasticity [99, 114]. PKCλ was found to phosphorylate Ser-818 on the GluA1 subunit which mediates PI3K-induced AMPAR insertion [115]. GluR2 can also regulate synaptic plasticity through Ser-880 phosphorylation-dependent interactions with PDZ domain-containing proteins, which regulate receptor internalization in the hippocampus [116] and cerebellum [117]. Thus, due to the actions of CRF on cAMP production and PKA activity, stress could potentially impact LC plasticity through modulation of AMPAR function which we have demonstrated in the past [49, 62].

Another modification of AMPARs is ubiquitination, which promotes endocytosis and degradation [108, 109, 118–120] and can occur at multiple places across the subunits. One effect of stress on AMPAR ubiquitination is to decrease glutamatergic synaptic transmission in the prefrontal cortex (PFC), which requires specific ubiquitin ligases Nedd4-1 and Fbx2, an effect which can be blocked by a proteasome inhibitor [121]. Identification of LC-specific ubiquitin ligases would help to find a precise target for stress response control and intervention. In contrast to ubiquitination, AMPAR C-terminal cysteine residue palmitoylation protects from degradation [122] and regulates its internalization [123]. Palmitoylation of the transmembrane domain promotes accumulation of AMPAR in the Golgi, possibly performing a quality control step for proper folding, while depalmitoylation stimulates membrane insertion of AMPAR [123–125]. Identifying any potential mechanisms linking CRF signaling to AMPAR ubiquitination in LC or elsewhere would be informative of means of promoting or inhibiting stress-induced plasticity within the nucleus.

### 2.4. CRF and Intracellular Signal Transduction

CRF mediates its action through CRFR1, which through Gs-coupled mechanisms increases the concentration of cAMP that phosphorylates PKA [52]. However, there is another mechanism caused by CRFR1 activation which acts through a MAPK cascade, in which a Gq-coupled mechanism increases concentration of phospholipase C (PLC), which activates metabolites which phosphorylate PKC, which in turn phosphorylates ERK1/2 [50, 126]. CRF has been shown to cause an increase in LC neurite length, an effect that is abolished by specific inhibition of PKA or MAPK, but not PKC [50]. PKC appears to also trigger a RhoA-activating cascade through downstream Rho-associated protein kinase (ROCK), which subsequently phosphorylates the collapsin response mediator protein 2 (CRMP-2) and causes growth cone collapse [127] (Figure 2).

Such receptor-mediated acute cellular changes could occur through the aforementioned mechanisms, but long-term changes could also potentially occur through regulation of gene expression. A study of single and repeated restraint stress demonstrated an increase in immunoreactivity of c-Fos, pERK, pCaMKII, and pCREB in the LC two hours following the stressor [128]. The same study also showed that pERK and pCREB had the same expression pattern and were colocalized to the same neurons, suggesting that activation of the MAPK/ERK pathways with CREB phosphorylation promote changes in gene expression. The exact mechanism of transcriptional changes following CRF expression in the LC is not clear. However, another study demonstrated increased c-Fos expression and CREB phosphorylation after acute immobilization stress, while repeated stress increased phosphorylation of p38, cJun N-teminal kinase (JNK 1/2/3), and ERK1/2 [129]. CREB is a transcriptional factor which regulates transcription of multiple downstream genes including c-Fos, brain-derived neurotrophic factor (BDNF), tyrosine hydroxylase (TH), and neuropeptides [130, 131]. These observations corroborate other studies that have shown altered expression of trophic factors and NE synthetic enzymes [18, 28, 54–57, 61].

BDNF stimulates dendritic outgrowth and increased synaptic connectivity. Mice genetically overexpressing the receptor for neurotrophin 3, TrkC, show increased anxiety-like behavior, as well as increased LC neuronal density [132], suggesting that some degree of positive feedback might exist between stress, neurotrophin 3 signaling, and LC plasticity. This is particularly interesting in light of preliminary observations by our laboratory that show that one week after acute stressor exposure, neurotrophin mRNA is increased, an effect accompanied by a trend for increased LC dendritic length and complexity (Figure 2). Others have also reported increased neurotrophin 3 expression in LC following stress, which can be normalized with antidepressant treatment [18]. Interestingly, in addition to sexual dimorphism of LC morphology and stress responsiveness, there are also sex differences in LC intracellular signaling induced by CRF: specifically, CRFR1 is more strongly coupled to *β*-arrestin in males, promoting receptor internalization and potentially blunted responsiveness to CRF in the future. In females, however, CRFR1 is more strongly coupled to Gs signaling pathways, which promotes increased LC discharge and dendritic proliferation, potentially increasing future sensitivity to stress by providing more space for synaptic contacts with CRF-positive afferents [63, 133]. In this way, the male and female LC may be differentially aligned to respond to stress with specific neuroplastic adaptations that promote disease.

## 3. CNS Neural Plasticity Changes in Response to NE Volume Transmission Changes

LC contributes to major CNS functions such as waking, arousal, attention, sensory discrimination, and cognition. Because stress promotes both short- and long-term functional and neuroplastic changes in LC, some of these functions might also be impacted by stressor exposure, either directly or indirectly. Through modulation of intrinsic and synaptic features of LC neurons, stress likely modifies noradrenergic volume transmission in target brain areas such as the amygdala, hippocampus, and PFC, where it potently modulates neural plasticity [134–138]. Because stress acutely and chronically alters LC discharge [53, 62, 66, 86] and NE release [78–82], different adrenergic receptors might become engaged during and after stressor exposure. The adrenergic receptors vary in their affinity for NE [3, 78, 139, 140], and different receptors promote different forms of plasticity and learning [134, 135, 137, 141]. Low concentrations of NE engage the high-affinity *α*_1_ receptor, particularly in the prefrontal cortex, which promotes working memory, sustained attention, and other cognitive functions [3, 142, 143].

Conversely, high NE concentration which occurs in response to stress causes engagement of the *α*_1_ and *β* adrenergic receptors. The *α*_1_ receptor promotes LTD of prefrontal synapses [137] and inhibition of prefrontal-dependent cognitive functions such as working memory and sustained attention [2, 83]. Indeed, enhanced *α*_1_ signaling in PFC is associated with increased behavioral flexibility [144]. Furthermore, stressor exposure has been shown to increase tonic LC discharge and promote scanning attention and behavioral flexibility [65, 66]. It has been proposed that such a change would permit lower-order sensorimotor regions to guide behavior with little modulation by prefrontal circuitry, allowing disengagement from specific stimuli and goal-oriented behaviors to instead promote rapid impulsive responses [2]. Such a stress-induced shift might be beneficial when an animal is faced with a threatening stimulus and a quick escape must be made. Additionally, engagement of the *β* receptor promotes hippocampal plasticity and encoding and recall of contextual fear memory [141, 145, 146]. Therefore, persistent stress-induced changes in LC function would elevate NE concentration in prefrontal cortex and hippocampus enhancing plasticity in both areas through signaling at *α*_1_ and *β* receptors to synergistically promote encoding and recall of fear memories, impaired cognition, hypervigilance, and behaviors that allow an appropriate behavioral response to be generated. Therefore, an inverted U relationship between LC firing and arousal/behavioral performance model has been proposed [2, 37, 139], with maximal cognitive function corresponding to “ideal” levels of LC tonic firing [147] and hyperarousal and vigilance corresponding with excessive levels of discharge. During stress, increased tonic LC firing is enhanced, which leads to increased levels of NE in LC projection fields, promoting broad scanning attention, hyperarousal, hypervigilance, and other anxiety-like behavioral symptoms in stressed subjects.

## 4. Role of Stress-Induced LC/NE Changes in PTSD

Chronic stress-induced alterations in LC structure and function that lead to behavioral impairments might contribute to disease pathogenesis and symptomatology. Many studies show the involvement or potential involvement of the LC in stress-related disease states, particularly PTSD. Both peripheral and central measures of NE activity are increased in PTSD patient populations, including enhanced sympathetic nervous system function [148–151], and increased functional connectivity between LC and the basolateral amygdala during conscious processing of threatening stimuli [152]. This enhanced connectivity is particularly important because of the role that the basolateral amygdala and its noradrenergic inputs in particular [153–157] play in fear conditioning. Furthermore, PTSD patients frequently show disturbances in sleep patterns [158–160], which may be related to chronically elevated LC discharge due to its well-established role in mediating arousal and a forebrain EEG associated with waking [161]. Such an effect could potentially be related to dysregulation of other stress-sensitive systems, such as the HPA axis which releases CRF. LC is potently activated by CRF, and increased levels of the peptide have been found in the cerebrospinal fluid of combat veterans afflicted with PTSD [162, 163], providing a potential means for maintaining LC hyperactivity even in the absence of a stressor. More recently, an fMRI study showed that PTSD patients showed exaggerated behavioral and autonomic responses to loud sounds, suggesting sensitized phasic responses of LC neurons [34]. Evidence for LC as a central mediator of PTSD-like symptoms comes from observations that yohimbine, an *α*_2A_ receptor antagonist which disinhibits LC neurons, produces panic attacks in up to 70% of PTSD patients and in 89% of patients with comorbid PTSD and panic disorder, but not in control subjects. Additionally, plasma levels of a NE metabolite postyohimbine administration were twice as high in PTSD patients [163]. These findings suggest that NE release is altered presynaptically at the level of the LC in PTSD patients, which may affect many downstream targets [164].

In contrast to the actions of yohimbine, clonidine, a presynaptic *α*_2A_ receptor agonist which limits noradrenergic transmission in the forebrain, has been shown to have beneficial effects on hyperarousal, hypervigilance, sleep disruption, exaggerated startle responses, and nightmares in veterans with PTSD [31]. The notion that increased NE release promotes some behaviors associated with PTSD and anxiety is further supported by observations that the *β*-receptor antagonist propranolol attenuates PTSD symptoms, possibly due to the actions that the *β* receptor plays in fear memory consolidation and emotion [31, 134, 135, 165–167]. Prazosin, an *α*_1_-adrenergic antagonist, has also been shown to be beneficial for alleviating nightmares and sleep disturbances in both veteran [168] and children PTSD patients [169], as well as for improving symptoms of hyperarousal, avoidance/numbing, and traumatic recall of past events [170]. It is also interesting to note that an *in vivo* PET study that the availability of the NE transporter in the LC is decreased in PTSD patients [171]. This could be indicative of elevated extracellular NE concentration and would be consistent with other reports of LC hyperactivity in this population. Thus, due to the well-documented ability of stress to promote forebrain NE release through short-term physiological activation and enduring molecular and cellular changes in the LC, stress-induced neuroplastic adaptations in the LC likely contribute to disease pathogenesis. This could occur at the level of the LC as a primary site of stress-induced plasticity or in downstream targets of the LC due to the well-established role of NE in mediating neuroplastic changes throughout the brain: fMRI studies have also shown changes in hippocampal volume and altered function in the amygdala, hippocampus, mPFC, orbitofrontal cortex, anterior cingulate, and insular cortex in PTSD patients [172], all of which may be related to maladaptive plastic changes in the LC or the plastic changes promoted by it [134, 135, 137, 165].

Based on these clinical reports, there is clear evidence that LC hyperfunction is at least characteristic of, if not causal to, PTSD symptomatology. However, some clinical observations suggest a more complicated relationship that exists between LC function and PTSD disease progression and treatment. As mentioned above, there is clinical evidence for decreased NE transporter availability in the LC of PTSD patients. This could be explained by elevated extracellular NE concentration; another potential explanation could be LC neuronal loss. Indeed, a postmortem neuromorphometric analysis of veterans with probable or possible war-related PTSD showed a lower LC cell count compared to controls [173], suggesting that the LC plays in the role in the pathophysiology of PTSD, or that a lower LC cell count may predispose individuals to PTSD. While decreased LC cell numbers would suggest reduced forebrain NE levels, in other pathologies in which LC cell counts are decreased such as Alzheimer's disease, the surviving neurons show evidence for hyperactivity [174] and dendritic sprouting and remodeling [107]. Additionally, recent clinical trials using 3,4-methylenedioxymethamphetamine (MDMA) have shown promising results in reduction or remission of PTSD symptoms: specifically, six phase II clinical trials have shown that combined MDMA and psychotherapy are safe and efficacious such that 52.7% of patients receiving active drug no longer meet PTSD criteria [175]. Despite a wealth of evidence showing that enhanced noradrenergic transmission contributes to PTSD etiology, MDMA increases release of catecholamines, including NE. One potential explanation for MDMA's somewhat paradoxical efficacy is that memory reconsolidation is enhanced via plastic changes in the hippocampus due to elevated NE levels [141, 145]. MDMA also facilitates fear extinction learning [176], and thus, enhanced NE efflux following MDMA administration might also promote plastic changes within the amygdala [12]. Additionally, because NE is generally increased in PTSD patients, the benefits of MDMA on symptom improvement are likely due to the drug complex polypharmacological interactions and its effects on brainwide neurotransmission. It is hypothesized that in addition to enhanced plasticity and memory reconsolidation, heightened monoaminergic neurotransmission following MDMA administration promotes a number of subjective psychological effects such as increased introspection and receptiveness for psychotherapy that lead to improved outcomes in PTSD patients [175]. Collectively, however, many clinical observations strongly suggest that hyperactive noradrenergic transmission contributes to PTSD symptomology and anxiety-like behavior.

## 5. Conclusions

Stressor exposure induces a series of neuroendocrine, physiological, and behavioral adaptations that promote an appropriate response to the stressor. Central to these diverse functions is CRF signaling which in a number of brain regions promotes a number of immediate [48, 49, 51, 177–184] and persistent [50, 52, 60, 121, 185–187] cellular changes. These effects are of particular interest in LC, where the interaction of CRF with its receptor CRFR1 activates cAMP-dependent intracellular signaling cascades, increasing tonic discharge and promoting anxiety-like behavior [64, 77, 188, 189]. Evidence suggests that chronic stressor exposure is able to alter LC gene expression [18, 28, 54–57, 61], promote long-term changes in synaptic transmission and excitability [53, 62] and receptor trafficking [58–60, 185], and, importantly, induce morphological changes and dendritic remodeling (Figure 1) [50, 52, 53, 57, 190]. These actions appear to be dependent on a number of kinases and GTPases and their associated signaling pathways [50, 52, 57] and potentially on AMPAR function [191, 192] which is modulated by CRF in the short term [49] and stressor exposure in the long term [62]. Through its complex signaling cascades, CRFR1 activation in LC induces a number of long-lasting cellular effects which ultimately impact the function of the nucleus itself as well as other target brain regions which are heavily innervated by LC and modulated by noradrenergic transmission. Critically, the LC promotes plasticity in other structures including the PFC, amygdala, and hippocampus by promoting noradrenergic transmission at *α*_1_ and *β* receptors [137, 141, 145, 146]. Therefore, stress and CRF can induce neuroplastic changes in LC, which can lead to subsequent neuroplastic changes elsewhere, ultimately promoting causing chronic anxiety-like behavior. Specifically, increased tonic discharge in the short term will drive an animal to display such behavior [62, 64, 77]. Maintenance of increased LC discharge in the long term [62] along with enhanced expression of genes necessary for NE synthesis and release [54–56] will lead to chronically elevated forebrain NE levels. This promotes network adaptations and plasticity in target regions which facilitate fear memory encoding and drive an animal towards a behavioral state characterized by vigilance, impulsivity, and impaired cognition [3, 78, 83, 193]. Meanwhile, morphological plasticity and dendritic outgrowth into the peri-LC area [50, 52, 53, 68, 194] will make LC subject to greater afferent regulation by stress-responsive structures such as PVN and CeA [58, 63, 194]. Through these mechanisms, chronic or traumatic stress could permanently alter forebrain noradrenergic transmission to promote long-lasting changes in behavior, manifesting in humans as mood and anxiety disorders such as depression and posttraumatic stress disorder. Thus, identifying how stress and CRF promote synaptic and morphological plasticity in LC to chronically elevate forebrain NE concentration represents an important step in understanding disease pathogenesis and symptomatology for mood, anxiety, and other neuropsychiatric disorders.

## References

[1] Greenberg P. E., Sisitsky T., Kessler R. C. (1999). The economic burden of anxiety disorders in the 1990s. *The Journal of Clinical Psychiatry*.

[2] Arnsten A. F. T. (2000). Through the looking glass: differential noradenergic modulation of prefrontal cortical function. *Neural Plasticity*.

[3] Arnsten A. F. T. (2011). Prefrontal cortical network connections: key site of vulnerability in stress and schizophrenia. *International Journal of Developmental Neuroscience*.

[4] Crupi R., Marino A., Cuzzocrea S. (2011). New therapeutic strategy for mood disorders. *Current Medicinal Chemistry*.

[5] Curtis G. C., Abelson J. L., Gold P. W. (1997). Adrenocorticotropic hormone and cortisol responses to corticotropin-releasing hormone: changes in panic disorder and effects of alprazolam treatment. *Biological Psychiatry*.

[6] Deakin J. F. W. (1991). Depression and 5HT. *International Clinical Psychopharmacology*.

[7] Ho J., Ngai S. P. C., Wu W. K. K., Hou W. K. (2018). Association between daily life experience and psychological well-being in people living with nonpsychotic mental disorders: protocol for a systematic review and meta-analysis. *Medicine*.

[8] Tay T. L., Béchade C., D’Andrea I. (2018). Microglia gone rogue: impacts on psychiatric disorders across the lifespan. *Frontiers in Molecular Neuroscience*.

[9] Wood S. K., Walker H. E., Valentino R. J., Bhatnagar S. (2010). Individual differences in reactivity to social stress predict susceptibility and resilience to a depressive phenotype: role of corticotropin-releasing factor. *Endocrinology*.

[10] Wood C. S., Valentino R. J., Wood S. K. (2017). Individual differences in the locus coeruleus-norepinephrine system: relevance to stress-induced cardiovascular vulnerability. *Physiology & Behavior*.

[11] Berton O., McClung C., Dileone R. J. (2006). Essential role of BDNF in the mesolimbic dopamine pathway in social defeat stress. *Science*.

[12] Bremner J. D., Elzinga B., Schmahl C., Vermetten E. (2008). Structural and functional plasticity of the human brain in posttraumatic stress disorder. *Progress in Brain Research*.

[13] Campos A. C., Ferreira F. R., da Silva W. A., Guimarães F. S. (2013). Predator threat stress promotes long lasting anxiety-like behaviors and modulates synaptophysin and CB1 receptors expression in brain areas associated with PTSD symptoms. *Neuroscience Letters*.

[14] Challis C., Boulden J., Veerakumar A. (2013). Raphe GABAergic neurons mediate the acquisition of avoidance after social defeat. *Journal of Neuroscience*.

[15] Chocyk A., Bobula B., Dudys D. (2013). Early-life stress affects the structural and functional plasticity of the medial prefrontal cortex in adolescent rats. *European Journal of Neuroscience*.

[16] Krishnan V., Han M. H., Graham D. L. (2007). Molecular adaptations underlying susceptibility and resistance to social defeat in brain reward regions. *Cell*.

[17] Kuipers S. D., Trentani A., den Boer J. A., ter Horst G. J. (2003). Molecular correlates of impaired prefrontal plasticity in response to chronic stress. *Journal of Neurochemistry*.

[18] Smith M. A., Makino S., Altemus M. (1995). Stress and antidepressants differentially regulate neurotrophin 3 mRNA expression in the locus coeruleus. *Proceedings of the National Academy of Sciences of the United States of America*.

[19] Veerakumar A., Challis C., Gupta P. (2014). Antidepressant-like effects of cortical deep brain stimulation coincide with pro-neuroplastic adaptations of serotonin systems. *Biological Psychiatry*.

[20] Zoladz P. R., Park C. R., Halonen J. D. (2012). Differential expression of molecular markers of synaptic plasticity in the hippocampus, prefrontal cortex, and amygdala in response to spatial learning, predator exposure, and stress-induced amnesia. *Hippocampus*.

[21] Jeanneteau F., Barrère C., Vos M. (2018). The stress-induced transcription factor NR4A1 adjusts mitochondrial function and synapse number in prefrontal cortex. *The Journal of Neuroscience*.

[22] Scarante F. F., Vila-Verde C., Detoni V. L., Ferreira-Junior N. C., Guimarães F. S., Campos A. C. (2017). Cannabinoid modulation of the stressed hippocampus. *Frontiers in Molecular Neuroscience*.

[23] Bath K. G., Russo S. J., Pleil K. E., Wohleb E. S., Duman R. S., Radley J. J. (2017). Circuit and synaptic mechanisms of repeated stress: perspectives from differing contexts, duration, and development. *Neurobiology of Stress*.

[24] Nikolova Y. S., Misquitta K. A., Rocco B. R. (2018). Shifting priorities: highly conserved behavioral and brain network adaptations to chronic stress across species. *Translational Psychiatry*.

[25] Paul I. A., Skolnick P. (2003). Glutamate and depression: clinical and preclinical studies. *Annals of the New York Academy of Sciences*.

[26] Lucassen P. J., Pruessner J., Sousa N. (2014). Neuropathology of stress. *Acta Neuropathologica*.

[27] Leonard B. E. (2007). Psychopathology of depression. *Drugs of Today*.

[28] Mamalaki E., Kvetnansky R., Brady L. S., Gold P. W., Herkenham M. (1992). Repeated immobilization stress alters tyrosine hydroxylase, corticotropin-releasing hormone and corticosteroid receptor messenger ribonucleic acid levels in rat brain. *Journal of Neuroendocrinology*.

[29] Weinshenker D. (2008). Functional consequences of locus coeruleus degeneration in Alzheimer’s disease. *Current Alzheimer Research*.

[30] Bremner J. D. (2006). Traumatic stress: effects on the brain. *Dialogues in Clinical Neuroscience*.

[31] Strawn J. R., Geracioti T. D. (2008). Noradrenergic dysfunction and the psychopharmacology of posttraumatic stress disorder. *Depression and Anxiety*.

[32] Borodovitsyna O., Flamini M., Chandler D. (2017). Noradrenergic modulation of cognition in health and disease. *Neural Plasticity*.

[33] Eser R. A., Ehrenberg A. J., Petersen C. (2018). Selective vulnerability of brainstem nuclei in distinct tauopathies: a postmortem study. *Journal of Neuropathology and Experimental Neurology*.

[34] Naegeli C., Zeffiro T., Piccirelli M. (2018). Locus coeruleus activity mediates hyperresponsiveness in posttraumatic stress disorder. *Biological Psychiatry*.

[35] Cunningham E. T., Bohn M. C., Sawchenko P. E. (1990). Organization of adrenergic inputs to the paraventricular and supraoptic nuclei of the hypothalamus in the rat. *The Journal of Comparative Neurology*.

[36] Herman J. P., Figueiredo H., Mueller N. K. (2003). Central mechanisms of stress integration: hierarchical circuitry controlling hypothalamo–pituitary–adrenocortical responsiveness. *Frontiers in Neuroendocrinology*.

[37] Berridge C. W., Waterhouse B. D. (2003). The locus coeruleus–noradrenergic system: modulation of behavioral state and state-dependent cognitive processes. *Brain Research Reviews*.

[38] Chandler D. J., Gao W. J., Waterhouse B. D. (2014). Heterogeneous organization of the locus coeruleus projections to prefrontal and motor cortices. *Proceedings of the National Academy of Sciences of the United States of America*.

[39] Foote S. L., Bloom F. E., Aston-Jones G. (1983). Nucleus locus ceruleus: new evidence of anatomical and physiological specificity. *Physiological Reviews*.

[40] Loughlin S. E., Foote S. L., Bloom F. E. (1986). Efferent projections of nucleus locus coeruleus: topographic organization of cells of origin demonstrated by three-dimensional reconstruction. *Neuroscience*.

[41] Robertson S. D., Plummer N. W., de Marchena J., Jensen P. (2013). Developmental origins of central norepinephrine neuron diversity. *Nature Neuroscience*.

[42] Chandler D., Waterhouse B. D. (2012). Evidence for broad versus segregated projections from cholinergic and noradrenergic nuclei to functionally and anatomically discrete subregions of prefrontal cortex. *Frontiers in Behavioral Neuroscience*.

[43] Chandler D. J. (2016). Evidence for a specialized role of the locus coeruleus noradrenergic system in cortical circuitries and behavioral operations. *Brain Research*.

[44] Chandler D. J., Lamperski C. S., Waterhouse B. D. (2013). Identification and distribution of projections from monoaminergic and cholinergic nuclei to functionally differentiated subregions of prefrontal cortex. *Brain Research*.

[45] Chandler D. J., Waterhouse B. D., Gao W. J. (2014). New perspectives on catecholaminergic regulation of executive circuits: evidence for independent modulation of prefrontal functions by midbrain dopaminergic and noradrenergic neurons. *Frontiers in Neural Circuits*.

[46] Devilbiss D. M., Page M. E., Waterhouse B. D. (2006). Locus ceruleus regulates sensory encoding by neurons and networks in waking animals. *Journal of Neuroscience*.

[47] Waterhouse B. D., Moises H. C., Woodward D. J. (1998). Phasic activation of the locus coeruleus enhances responses of primary sensory cortical neurons to peripheral receptive field stimulation. *Brain Research*.

[48] Jedema H. P., Grace A. A. (2004). Corticotropin-releasing hormone directly activates noradrenergic neurons of the locus ceruleus recorded *in vitro*. *Journal of Neuroscience*.

[49] Prouty E. W., Waterhouse B. D., Chandler D. J. (2017). Corticotropin releasing factor dose-dependently modulates excitatory synaptic transmission in the noradrenergic nucleus locus coeruleus. *European Journal of Neuroscience*.

[50] Swinny J. D., Valentino R. J. (2006). Corticotropin-releasing factor promotes growth of brain norepinephrine neuronal processes through Rho GTPase regulators of the actin cytoskeleton in rat. *European Journal of Neuroscience*.

[51] Valentino R. J., Foote S. L., Aston-Jones G. (1983). Corticotropin-releasing factor activates noradrenergic neurons of the locus coeruleus. *Brain Research*.

[52] Cibelli G., Corsi P., Diana G., Vitiello F., Thiel G. (2001). Corticotropin-releasing factor triggers neurite outgrowth of a catecholaminergic immortalized neuron via cAMP and MAP kinase signalling pathways. *European Journal of Neuroscience*.

[53] Swinny J. D., O’Farrell E., Bingham B. C., Piel D. A., Valentino R. J., Beck S. G. (2010). Neonatal rearing conditions distinctly shape locus coeruleus neuronal activity, dendritic arborization, and sensitivity to corticotrophin-releasing factor. *The International Journal of Neuropsychopharmacology*.

[54] Fan Y., Chen P., Li Y., Zhu M. Y. (2013). Effects of chronic social defeat on expression of dopamine *β*-hydroxylase in rat brains. *Synapse*.

[55] Rusnák M., Kvetňanský R., Jeloková J., Palkovits M. (2001). Effect of novel stressors on gene expression of tyrosine hydroxylase and monoamine transporters in brainstem noradrenergic neurons of long-term repeatedly immobilized rats. *Brain Research*.

[56] George S. A., Knox D., Curtis A. L., Aldridge J. W., Valentino R. J., Liberzon I. (2013). Altered locus coeruleus–norepinephrine function following single prolonged stress. *European Journal of Neuroscience*.

[57] Salim S., Hite B., Eikenburg D. C. (2007). Activation of the CRF_1_ receptor causes ERK1/2 mediated increase in GRK3 expression in CATH.a cells. *FEBS Letters*.

[58] Bangasser D. A., Reyes B. A. S., Piel D. (2013). Increased vulnerability of the brain norepinephrine system of females to corticotropin-releasing factor overexpression. *Molecular Psychiatry*.

[59] Reyes B. A. S., Bangasser D. A., Valentino R. J., Van Bockstaele E. J. (2014). Using high resolution imaging to determine trafficking of corticotropin-releasing factor receptors in noradrenergic neurons of the rat locus coeruleus. *Life Sciences*.

[60] Reyes B. A. S., Valentino R. J., Van Bockstaele E. J. (2008). Stress-induced intracellular trafficking of corticotropin-releasing factor receptors in rat locus coeruleus neurons. *Endocrinology*.

[61] Sabban E. L., Serova L. I., Newman E., Aisenberg N., Akirav I. (2018). Changes in gene expression in the locus coeruleus-amygdala circuitry in inhibitory avoidance PTSD model. *Cellular and Molecular Neurobiology*.

[62] Borodovitsyna O., Flamini M. D., Chandler D. J. (2018). Acute stress persistently alters locus coeruleus function and anxiety-like behavior in adolescent rats. *Neuroscience*.

[63] Valentino R. J., Bangasser D., Van Bockstaele E. J. (2013). Sex-biased stress signaling: the corticotropin-releasing factor receptor as a model. *Molecular Pharmacology*.

[64] McCall J. G., al-Hasani R., Siuda E. R. (2015). CRH engagement of the locus coeruleus noradrenergic system mediates stress-induced anxiety. *Neuron*.

[65] Curtis A. L., Leiser S. C., Snyder K., Valentino R. J. (2012). Predator stress engages corticotropin-releasing factor and opioid systems to alter the operating mode of locus coeruleus norepinephrine neurons. *Neuropharmacology*.

[66] Snyder K., Wang W. W., Han R., McFadden K., Valentino R. J. (2012). Corticotropin-releasing factor in the norepinephrine nucleus, locus coeruleus, facilitates behavioral flexibility. *Neuropsychopharmacology*.

[67] Schwarz L. A., Miyamichi K., Gao X. J. (2015). Viral-genetic tracing of the input–output organization of a central noradrenaline circuit. *Nature*.

[68] Bangasser D. A., Zhang X., Garachh V., Hanhauser E., Valentino R. J. (2011). Sexual dimorphism in locus coeruleus dendritic morphology: a structural basis for sex differences in emotional arousal. *Physiology & Behavior*.

[69] Kreibich A., Reyes B. A. S., Curtis A. L. (2008). Presynaptic inhibition of diverse afferents to the locus ceruleus by *κ*-opiate receptors: a novel mechanism for regulating the central norepinephrine system. *Journal of Neuroscience*.

[70] Reyes B. A. S., Valentino R. J., Xu G., Van Bockstaele E. J. (2005). Hypothalamic projections to locus coeruleus neurons in rat brain. *European Journal of Neuroscience*.

[71] Reyes B. A. S., Zitnik G., Foster C., Van Bockstaele E. J., Valentino R. J. (2015). Social stress engages neurochemically-distinct afferents to the rat locus coeruleus depending on coping strategy. *eNeuro*.

[72] Valentino R. J., Rudoy C., Saunders A., Liu X. B., van Bockstaele E. J. (2001). Corticotropin-releasing factor is preferentially colocalized with excitatory rather than inhibitory amino acids in axon terminals in the peri-locus coeruleus region. *Neuroscience*.

[73] Valentino R. J., Page M., van Bockstaele E., Aston-Jones G. (1992). Corticotropin-releasing factor innervation of the locus coeruleus region: distribution of fibers and sources of input. *Neuroscience*.

[74] Aston-Jones G., Cohen J. D. (2005). Adaptive gain and the role of the locus coeruleus–norepinephrine system in optimal performance. *The Journal of Comparative Neurology*.

[75] Bangasser D. A., Valentino R. J. (2014). Sex differences in stress-related psychiatric disorders: neurobiological perspectives. *Frontiers in Neuroendocrinology*.

[76] Valentino R. J., Foote S. L., Page M. E. (1993). The locus coeruleus as a site for integrating corticotropin-releasing factor and noradrenergic mediation of stress responses. *Annals of the New York Academy of Sciences*.

[77] McCall J. G., Siuda E. R., Bhatti D. L. (2017). Locus coeruleus to basolateral amygdala noradrenergic projections promote anxiety-like behavior. *eLife*.

[78] Arnsten A. F. T. (1998). Catecholamine modulation of prefrontal cortical cognitive function. *Trends in Cognitive Sciences*.

[79] Rajbhandari A. K., Baldo B. A., Bakshi V. P. (2015). Predator stress-induced CRF release causes enduring sensitization of basolateral amygdala norepinephrine systems that promote PTSD-like startle abnormalities. *Journal of Neuroscience*.

[80] Bouchez G., Millan M. J., Rivet J. M., Billiras R., Boulanger R., Gobert A. (2012). Quantification of extracellular levels of corticosterone in the basolateral amygdaloid complex of freely-moving rats: a dialysis study of circadian variation and stress-induced modulation. *Brain Research*.

[81] Patki G., Atrooz F., Alkadhi I., Solanki N., Salim S. (2015). High aggression in rats is associated with elevated stress, anxiety-like behavior, and altered catecholamine content in the brain. *Neuroscience Letters*.

[82] Mana M. J., Grace A. A. (1997). Chronic cold stress alters the basal and evoked electrophysiological activity of rat locus coeruleus neurons. *Neuroscience*.

[83] Arnsten A. F. T., Mathew R., Ubriani R., Taylor J. R., Li B.-M. (1999). *α*-1 noradrenergic receptor stimulation impairs prefrontal cortical cognitive function. *Biological Psychiatry*.

[84] Korf J., Aghajanian G. K., Roth R. H. (1973). Increased turnover of norepinephrine in the rat cerebral cortex during stress: role of the locus coeruleus. *Neuropharmacology*.

[85] Bingham B., McFadden K., Zhang X., Bhatnagar S., Beck S., Valentino R. (2011). Early adolescence as a critical window during which social stress distinctly alters behavior and brain norepinephrine activity. *Neuropsychopharmacology*.

[86] Valentino R. J., Foote S. L. (1988). Corticotropin-releasing hormone increases tonic but not sensory-evoked activity of noradrenergic locus coeruleus neurons in unanesthetized rats. *The Journal of Neuroscience*.

[87] Lejeune F., Millan M. J. (2003). The CRF_1_ receptor antagonist, DMP695, abolishes activation of locus coeruleus noradrenergic neurones by CRF in anesthetized rats. *European Journal of Pharmacology*.

[88] Tashiro A., Yuste R. (2004). Regulation of dendritic spine motility and stability by Rac1 and Rho kinase: evidence for two forms of spine motility. *Molecular and Cellular Neuroscience*.

[89] Nakayama A. Y., Harms M. B., Luo L. (2000). Small GTPases Rac and Rho in the maintenance of dendritic spines and branches in hippocampal pyramidal neurons. *The Journal of Neuroscience*.

[90] Van Aelst L., Cline H. T. (2004). Rho GTPases and activity-dependent dendrite development. *Current Opinion in Neurobiology*.

[91] Van Bockstaele E. J., Reyes B. A. S., Valentino R. J. (2010). The locus coeruleus: a key nucleus where stress and opioids intersect to mediate vulnerability to opiate abuse. *Brain Research*.

[92] Liu Y., Nakamura S. (2006). Stress-induced plasticity of monoamine axons. *Frontiers in Bioscience*.

[93] Nakamura S., Sakaguchi T., Aoki F. (1989). Electrophysiological evidence for terminal sprouting of locus coeruleus neurons following repeated mild stress. *Neuroscience Letters*.

[94] Rasmusson A. M., Hauger R. L., Morgan C. A., Bremner J. D., Charney D. S., Southwick S. M. (2000). Low baseline and yohimbine-stimulated plasma neuropeptide Y (NPY) levels in combat-related PTSD. *Biological Psychiatry*.

[95] Reyes B. A. S., Glaser J. D., Van Bockstaele E. J. (2007). Ultrastructural evidence for co-localization of corticotropin-releasing factor receptor and *μ*-opioid receptor in the rat nucleus locus coeruleus. *Neuroscience Letters*.

[96] Van Bockstaele E. J., Colago E. E. O., Valentino R. J. (1998). Amygdaloid corticotropin-releasing factor targets locus coeruleus dendrites: substrate for the co-ordination of emotional and cognitive limbs of the stress response. *Journal of Neuroendocrinology*.

[97] Bangasser D. A., Eck S. R., Telenson A. M., Salvatore M. (2018). Sex differences in stress regulation of arousal and cognition. *Physiology & Behavior*.

[98] Kessler R. C., Berglund P., Demler O., Jin R., Merikangas K. R., Walters E. E. (2005). Lifetime prevalence and age-of-onset distributions of DSM-IV disorders in the National Comorbidity Survey Replication. *Archives of General Psychiatry*.

[99] Banke T. G., Bowie D., Lee H. K., Huganir R. L., Schousboe A., Traynelis S. F. (2000). Control of GluR1 AMPA receptor function by cAMP-dependent protein kinase. *The Journal of Neuroscience*.

[100] Esteban J. A., Shi S. H., Wilson C., Nuriya M., Huganir R. L., Malinow R. (2003). PKA phosphorylation of AMPA receptor subunits controls synaptic trafficking underlying plasticity. *Nature Neuroscience*.

[101] Roche K. W., O’Brien R. J., Mammen A. L., Bernhardt J., Huganir R. L. (1996). Characterization of multiple phosphorylation sites on the AMPA receptor GluR1 subunit. *Neuron*.

[102] Greger I. H., Watson J. F., Cull-Candy S. G. (2017). Structural and functional architecture of AMPA-type glutamate receptors and their auxiliary proteins. *Neuron*.

[103] Kato A. S., Witkin J. M. (2018). Auxiliary subunits of AMPA receptors: the discovery of a forebrain-selective antagonist, LY3130481/CERC-611. *Biochemical Pharmacology*.

[104] Kang M. G., Guo Y., Huganir R. L. (2009). AMPA receptor and GEF-H1/Lfc complex regulates dendritic spine development through RhoA signaling cascade. *Proceedings of the National Academy of Sciences of the United States of America*.

[105] Szíber Z., Liliom H., Morales C. O. O. (2017). Ras and Rab interactor 1 controls neuronal plasticity by coordinating dendritic filopodial motility and AMPA receptor turnover. *Molecular Biology of the Cell*.

[106] Gesi M., Soldani P., Giorgi F. S., Santinami A., Bonaccorsi I., Fornai F. (2000). The role of the locus coeruleus in the development of Parkinson’s disease. *Neuroscience & Biobehavioral Reviews*.

[107] McMillan P. J., White S. S., Franklin A. (2011). Differential response of the central noradrenergic nervous system to the loss of locus coeruleus neurons in Parkinson’s disease and Alzheimer’s disease. *Brain Research*.

[108] Lu W., Roche K. W. (2012). Posttranslational regulation of AMPA receptor trafficking and function. *Current Opinion in Neurobiology*.

[109] Widagdo J., Guntupalli S., Jang S. E., Anggono V. (2017). Regulation of AMPA receptor trafficking by protein ubiquitination. *Frontiers in Molecular Neuroscience*.

[110] Lussier M. P., Sanz-Clemente A., Roche K. W. (2015). Dynamic regulation of *N*-methyl-d-aspartate (NMDA) and *α*-amino-3-hydroxy-5-methyl-4-isoxazolepropionic acid (AMPA) receptors by posttranslational modifications. *Journal of Biological Chemistry*.

[111] Mammen A. L., Kameyama K., Roche K. W., Huganir R. L. (1997). Phosphorylation of the *α*-amino-3-hydroxy-5-methylisoxazole4-propionic acid receptor GluR1 subunit by calcium/calmodulin-dependent kinase II. *Journal of Biological Chemistry*.

[112] Jenkins M. A., Traynelis S. F. (2012). PKC phosphorylates GluA1-Ser831 to enhance AMPA receptor conductance. *Channels*.

[113] Jenkins M. A., Wells G., Bachman J. (2014). Regulation of GluA1 *α*-amino-3-hydroxy-5-methyl-4-isoxazolepropionic acid receptor function by protein kinase C at serine-818 and threonine-840. *Molecular Pharmacology*.

[114] Derkach V., Barria A., Soderling T. R. (1999). Ca^2+^/calmodulin-kinase II enhances channel conductance of *α*-amino-3-hydroxy-5-methyl-4-isoxazolepropionate type glutamate receptors. *Proceedings of the National Academy of Sciences of the United States of America*.

[115] Ren S. Q., Yan J. Z., Zhang X. Y. (2013). PKC*λ* is critical in AMPA receptor phosphorylation and synaptic incorporation during LTP. *The EMBO Journal*.

[116] Kim C. H., Chung H. J., Lee H. K., Huganir R. L. (2001). Interaction of the AMPA receptor subunit GluR2/3 with PDZ domains regulates hippocampal long-term depression. *Proceedings of the National Academy of Sciences of the United States of America*.

[117] Xia J., Chung H. J., Wihler C., Huganir R. L., Linden D. J. (2000). Cerebellar long-term depression requires PKC-regulated interactions between GluR2/3 and PDZ domain–containing proteins. *Neuron*.

[118] Hegde A. N. (2010). The ubiquitin-proteasome pathway and synaptic plasticity. *Learning & Memory*.

[119] Goo M. S., Scudder S. L., Patrick G. N. (2015). Ubiquitin-dependent trafficking and turnover of ionotropic glutamate receptors. *Frontiers in Molecular Neuroscience*.

[120] Jiang J., Suppiramaniam V., Wooten M. W. (2006). Posttranslational modifications and receptor-associated proteins in AMPA receptor trafficking and synaptic plasticity. *Neurosignals*.

[121] Yuen E. Y., Wei J., Liu W., Zhong P., Li X., Yan Z. (2012). Repeated stress causes cognitive impairment by suppressing glutamate receptor expression and function in prefrontal cortex. *Neuron*.

[122] Yang G., Xiong W., Kojic L., Cynader M. S. (2009). Subunit-selective palmitoylation regulates the intracellular trafficking of AMPA receptor. *European Journal of Neuroscience*.

[123] Hayashi T., Rumbaugh G., Huganir R. L. (2005). Differential regulation of AMPA receptor subunit trafficking by palmitoylation of two distinct sites. *Neuron*.

[124] Lin D. T., Makino Y., Sharma K. (2009). Regulation of AMPA receptor extrasynaptic insertion by 4.1N, phosphorylation and palmitoylation. *Nature Neuroscience*.

[125] Thomas G. M., Huganir R. L. (2013). Palmitoylation-dependent regulation of glutamate receptors and their PDZ domain-containing partners. *Biochemical Society Transactions*.

[126] Brar B. K., Chen A., Perrin M. H., Vale W. (2004). Specificity and regulation of extracellularly regulated kinase1/2 phosphorylation through corticotropin-releasing factor (CRF) receptors 1 and 2*β* by the CRF/urocortin family of peptides. *Endocrinology*.

[127] Arimura N., Menager C., Kawano Y. (2005). Phosphorylation by Rho kinase regulates CRMP-2 activity in growth cones. *Molecular and Cellular Biology*.

[128] Kwon M. S., Seo Y. J., Shim E. J., Choi S. S., Lee J. Y., Suh H. W. (2006). The effect of single or repeated restraint stress on several signal molecules in paraventricular nucleus, arcuate nucleus and locus coeruleus. *Neuroscience*.

[129] Hebert M. A., Serova L. I., Sabban E. L. (2005). Single and repeated immobilization stress differentially trigger induction and phosphorylation of several transcription factors and mitogen-activated protein kinases in the rat locus coeruleus. *Journal of Neurochemistry*.

[130] Nestler E. J. (2015). Chapter six—role of the brain’s reward circuitry in depression: transcriptional mechanisms. *International Review of Neurobiology*.

[131] Aurelian L., Warnock K. T., Balan I., Puche A., June H. (2016). TLR4 signaling in VTA dopaminergic neurons regulates impulsivity through tyrosine hydroxylase modulation. *Translational Psychiatry*.

[132] Dierssen M., Gratacòs M., Sahún I. (2006). Transgenic mice overexpressing the full-length neurotrophin receptor TrkC exhibit increased catecholaminergic neuron density in specific brain areas and increased anxiety-like behavior and panic reaction. *Neurobiology of Disease*.

[133] Zitnik G. A. (2016). Control of arousal through neuropeptide afferents of the locus coeruleus. *Brain Research*.

[134] Hansen N., Manahan-Vaughan D. (2015). Hippocampal long-term potentiation that is elicited by perforant path stimulation or that occurs in conjunction with spatial learning is tightly controlled by beta-adrenoreceptors and the locus coeruleus. *Hippocampus*.

[135] Hansen N., Manahan-Vaughan D. (2015). Locus coeruleus stimulation facilitates long-term depression in the dentate gyrus that requires activation of *β*-adrenergic receptors. *Cerebral Cortex*.

[136] Lemon N., Aydin-Abidin S., Funke K., Manahan-Vaughan D. (2009). Locus coeruleus activation facilitates memory encoding and induces hippocampal LTD that depends on *β*-adrenergic receptor activation. *Cerebral Cortex*.

[137] Marzo A., Bai J., Caboche J., Vanhoutte P., Otani S. (2010). Cellular mechanisms of long-term depression induced by noradrenaline in rat prefrontal neurons. *Neuroscience*.

[138] Salgado H., Trevino M., Atzori M. (2016). Layer- and area-specific actions of norepinephrine on cortical synaptic transmission. *Brain Research*.

[139] Arnsten A. F. T. (2007). Catecholamine and second messenger influences on prefrontal cortical networks of “representational knowledge”: a rational bridge between genetics and the symptoms of mental illness. *Cerebral Cortex*.

[140] Atzori M., Cuevas-Olguin R., Esquivel-Rendon E. (2016). Locus ceruleus norepinephrine release: a central regulator of CNS spatio-temporal activation?. *Frontiers in Synaptic Neuroscience*.

[141] Schutsky K., Ouyang M., Thomas S. A. (2011). Xamoterol impairs hippocampus-dependent emotional memory retrieval via G_i/o_-coupled *β*_2_-adrenergic signaling. *Learning & Memory*.

[142] Berridge C. W., Spencer R. C. (2016). Differential cognitive actions of norepinephrine a2 and a1 receptor signaling in the prefrontal cortex. *Brain Research*.

[143] Amemiya S., Kubota N., Umeyama N., Nishijima T., Kita I. (2016). Noradrenergic signaling in the medial prefrontal cortex and amygdala differentially regulates vicarious trial-and-error in a spatial decision-making task. *Behavioural Brain Research*.

[144] Nicholls R. E., Alarcon J. M., Malleret G. (2008). Transgenic mice lacking NMDAR-dependent LTD exhibit deficits in behavioral flexibility. *Neuron*.

[145] Murchison C. F., Zhang X. Y., Zhang W. P., Ouyang M., Lee A., Thomas S. A. (2004). A distinct role for norepinephrine in memory retrieval. *Cell*.

[146] Ouyang M., Young M. B., Lestini M. M., Schutsky K., Thomas S. A. (2012). Redundant catecholamine signaling consolidates fear memory via phospholipase C. *Journal of Neuroscience*.

[147] Howells F. M., Stein D. J., Russell V. A. (2012). Synergistic tonic and phasic activity of the locus coeruleus norepinephrine (LC-NE) arousal system is required for optimal attentional performance. *Metabolic Brain Disease*.

[148] Orr S. P., Lasko N. B., Metzger L. J., Pitman R. K. (1997). Physiologic responses to non-startling tones in Vietnam veterans with post-traumatic stress disorder. *Psychiatry Research*.

[149] Orr S. P., Lasko N. B., Shalev A. Y., Pitman R. K. (1995). Physiologic responses to loud tones in Vietnam veterans with posttraumatic stress disorder. *Journal of Abnormal Psychology*.

[150] Orr S. P., Metzger L. J., Lasko N. B. (2003). Physiologic responses to sudden, loud tones in monozygotic twins discordant for combat exposure: association with posttraumatic stress disorder. *Archives of General Psychiatry*.

[151] Pitman R. K., Orr S. P. (1990). Twenty-four hour urinary cortisol and catecholamine excretion in combat-related posttraumatic stress disorder. *Biological Psychiatry*.

[152] Lanius R. A., Rabellino D., Boyd J. E., Harricharan S., Frewen P. A., McKinnon M. C. (2017). The innate alarm system in PTSD: conscious and subconscious processing of threat. *Current Opinion in Psychology*.

[153] Soya S., Shoji H., Hasegawa E. (2013). Orexin receptor-1 in the locus coeruleus plays an important role in cue-dependent fear memory consolidation. *Journal of Neuroscience*.

[154] Uematsu A., Tan B. Z., Johansen J. P. (2015). Projection specificity in heterogeneous locus coeruleus cell populations: implications for learning and memory. *Learning & Memory*.

[155] Uematsu A., Tan B. Z., Ycu E. A. (2017). Modular organization of the brainstem noradrenaline system coordinates opposing learning states. *Nature Neuroscience*.

[156] Giustino T. F., Fitzgerald P. J., Maren S. (2016). Revisiting propranolol and PTSD: memory erasure or extinction enhancement?. *Neurobiology of Learning and Memory*.

[157] Giustino T. F., Maren S. (2018). Noradrenergic modulation of fear conditioning and extinction. *Frontiers in Behavioral Neuroscience*.

[158] Ross R. J., Ball W. A., Dinges D. F. (1994). Rapid eye movement sleep disturbance in posttraumatic stress disorder. *Biological Psychiatry*.

[159] Ross R. J., Ball W. A., Sanford L. D. (1999). Rapid eye movement sleep changes during the adaptation night in combat veterans with posttraumatic stress disorder. *Biological Psychiatry*.

[160] Mellman T. A., Kulick-Bell R., Ashlock L. E., Nolan B. (1995). Sleep events among veterans with combat-related posttraumatic stress disorder. *The American Journal of Psychiatry*.

[161] Page M. E., Berridge C. W., Foote S. L., Valentino R. J. (1993). Corticotropin-releasing factor in the locus coeruleus mediates EEG activation associated with hypotensive stress. *Neuroscience Letters*.

[162] Baker D. G., West S. A., Nicholson W. E. (1999). Serial CSF corticotropin-releasing hormone levels and adrenocortical activity in combat veterans with posttraumatic stress disorder. *American Journal of Psychiatry*.

[163] Bremner J. D., Innis R. B., Ng C. K. (1997). Positron emission tomography measurement of cerebral metabolic correlates of yohimbine administration in combat-related posttraumatic stress disorder. *Archives of General Psychiatry*.

[164] Fullerton C. S., Herberman Mash H. B., Benevides K. N., Morganstein J. C., Ursano R. J. (2015). Distress of routine activities and perceived safety associated with post-traumatic stress, depression, and alcohol use: 2002 Washington, DC, sniper attacks. *Disaster Medicine and Public Health Preparedness*.

[165] Hagena H., Hansen N., Manahan-Vaughan D. (2016). *β*-adrenergic control of hippocampal function: subserving the choreography of synaptic information storage and memory. *Cerebral Cortex*.

[166] Huang B., Zhu H., Zhou Y., Liu X., Ma L. (2017). Unconditioned- and conditioned- stimuli induce differential memory reconsolidation and *β*-AR-dependent CREB activation. *Frontiers in Neural Circuits*.

[167] Villain H., Benkahoul A., Birmes P., Ferry B., Roullet P. (2018). Influence of early stress on memory reconsolidation: implications for post-traumatic stress disorder treatment. *PLoS One*.

[168] Raskind M. A., Dobie D. J., Kanter E. D., Petrie E. C., Thompson C. E., Peskind E. R. (2000). The *α*_1_-adrenergic antagonist prazosin ameliorates combat trauma nightmares in veterans with posttraumatic stress disorder: a report of 4 cases. *The Journal of Clinical Psychiatry*.

[169] Keeshin B. R., Ding Q., Presson A. P., Berkowitz S. J., Strawn J. R. (2017). Use of prazosin for pediatric PTSD-associated nightmares and sleep disturbances: a retrospective chart review. *Neurology and Therapy*.

[170] Raskind M. A., Peskind E. R., Kanter E. D. (2003). Reduction of nightmares and other PTSD symptoms in combat veterans by prazosin: a placebo-controlled study. *American Journal of Psychiatry*.

[171] Pietrzak R. H., Gallezot J. D., Ding Y. S. (2013). Association of posttraumatic stress disorder with reduced in vivo norepinephrine transporter availability in the locus coeruleus. *JAMA Psychiatry*.

[172] Liberzon I., Phan K. L. (2003). Brain-imaging studies of posttraumatic stress disorder. *CNS Spectrums*.

[173] Bracha H. S., Garcia-Rill E., Mrak R. E., Skinner R. (2005). Postmortem locus coeruleus neuron count in three American veterans with probable or possible war-related PTSD. *The Journal of Neuropsychiatry and Clinical Neurosciences*.

[174] Hoogendijk W. J. G., Feenstra M. G. P., Botterblom M. H. A. (1999). Increased activity of surviving locus ceruleus neurons in Alzheimer’s disease. *Annals of Neurology*.

[175] Feduccia A. A., Mithoefer M. C. (2018). MDMA-assisted psychotherapy for PTSD: are memory reconsolidation and fear extinction underlying mechanisms?. *Progress in Neuro-Psychopharmacology & Biological Psychiatry*.

[176] Young M. B., Andero R., Ressler K. J., Howell L. L. (2015). 3,4-Methylenedioxymethamphetamine facilitates fear extinction learning. *Translational Psychiatry*.

[177] Curtis A. L., Lechner S. M., Pavcovich L. A., Valentino R. J. (1997). Activation of the locus coeruleus noradrenergic system by intracoerulear microinfusion of corticotropin-releasing factor: effects on discharge rate, cortical norepinephrine levels and cortical electroencephalographic activity. *Journal of Pharmacology and Experimental Therapeutics*.

[178] Asok A., Schulkin J., Rosen J. B. (2016). Corticotropin releasing factor type-1 receptor antagonism in the dorsolateral bed nucleus of the stria terminalis disrupts contextually conditioned fear, but not unconditioned fear to a predator odor. *Psychoneuroendocrinology*.

[179] Aston-Jones G., Shipley M. T., Chouvet G. (1991). Chapter 4 - afferent regulation of locus coeruleus neurons: anatomy, physiology and pharmacology. *Progress in Brain Research*.

[180] Koob G. F., Heinrichs S. C., Menzaghi F., Pich E. M., Britton K. T. (1994). Corticotropin releasing factor, stress and behavior. *Seminars in Neuroscience*.

[181] Kratzer S., Mattusch C., Metzger M. W. (2013). Activation of CRH receptor type 1 expressed on glutamatergic neurons increases excitability of CA1 pyramidal neurons by the modulation of voltage-gated ion channels. *Frontiers in Cellular Neuroscience*.

[182] Howerton A. R., Roland A. V., Fluharty J. M. (2014). Sex differences in corticotropin-releasing factor receptor-1 action within the dorsal raphe nucleus in stress responsivity. *Biological Psychiatry*.

[183] Kirby L. G., Freeman-Daniels E., Lemos J. C. (2008). Corticotropin-releasing factor increases GABA synaptic activity and induces inward current in 5-hydroxytryptamine dorsal raphe neurons. *Journal of Neuroscience*.

[184] Lamy C. M., Beck S. G. (2010). Swim stress differentially blocks CRF receptor mediated responses in dorsal raphe nucleus. *Psychoneuroendocrinology*.

[185] Holmes K. D., Babwah A. V., Dale L. B., Poulter M. O., Ferguson S. S. G. (2006). Differential regulation of corticotropin releasing factor 1*α* receptor endocytosis and trafficking by *β*-arrestins and Rab GTPases. *Journal of Neurochemistry*.

[186] Zhao-Shea R., DeGroot S. R., Liu L. (2015). Increased CRF signalling in a ventral tegmental area-interpeduncular nucleus-medial habenula circuit induces anxiety during nicotine withdrawal. *Nature Communications*.

[187] Gounko N. V., Swinny J. D., Kalicharan D. (2013). Corticotropin-releasing factor and urocortin regulate spine and synapse formation: structural basis for stress-induced neuronal remodeling and pathology. *Molecular Psychiatry*.

[188] Bangasser D. A. (2013). Sex differences in stress-related receptors: “micro” differences with “macro” implications for mood and anxiety disorders. *Biology of Sex Differences*.

[189] Graeff F. G. (1994). Neuroanatomy and neurotransmitter regulation of defensive behaviors and related emotions in mammals. *Brazilian Journal of Medical and Biological Research*.

[190] Widnell K. L., Chen J. S., Iredale P. A. (1996). Transcriptional regulation of CREB (cyclic AMP response element-binding protein) expression in CATH.a cells. *Journal of Neurochemistry*.

[191] Lussier M. P., Nasu-Nishimura Y., Roche K. W. (2011). Activity-dependent ubiquitination of the AMPA receptor subunit GluA2. *Journal of Neuroscience*.

[192] Malinow R., Malenka R. C. (2002). AMPA receptor trafficking and synaptic plasticity. *Annual Review of Neuroscience*.

[193] Aisa B., Tordera R., Lasheras B., del Río J., Ramírez M. J. (2007). Cognitive impairment associated to HPA axis hyperactivity after maternal separation in rats. *Psychoneuroendocrinology*.

[194] Valentino R. J., Reyes B., van Bockstaele E., Bangasser D. (2012). Molecular and cellular sex differences at the intersection of stress and arousal. *Neuropharmacology*.

